# The Quartile Levels of Thyroid-stimulating Hormone at Newborn Screening Stratified Risks of Neurodevelopmental Impairment in Extremely Preterm Infants: A Population Cohort Study

**DOI:** 10.2188/jea.JE20230253

**Published:** 2024-09-05

**Authors:** Li-Wen Chen, Chi-Hsiang Chu, Yung-Chieh Lin, Chao-Ching Huang

**Affiliations:** 1 Department of Pediatrics, National Cheng Kung University Hospital, College of Medicine, National Cheng Kung University, Tainan, Taiwan; 2 Department of Statistics, Tunghai University, Taichung, Taiwan; 3 Department of Pediatrics, College of Medicine, Taipei Medical University, Taipei, Taiwan

**Keywords:** thyroid-stimulating hormone, newborn screening, extremely preterm, neurodevelopmental outcome

## Abstract

**Background:**

To evaluate whether thyroid-stimulating hormone (TSH) measured during newborn screening (NBS) at birth and at discharge can be surrogate markers for neurodevelopmental impairment (NDI) in extremely preterm infants.

**Methods:**

The population cohort enrolled infants born <29 weeks’ gestation in 2008–2020 in southern Taiwan. Infants with a maternal history of thyroid disorders and infants who required thyroxine supplementation during hospitalization were excluded. TSH levels measured during NBS at birth and at term-equivalent age (TEA)/discharge were respectively categorized into the lowest quartile, the interquartile range, and the highest quartile, which were correlated to NDI outcomes.

**Results:**

Among 392 patients with paired TSH data, 358 (91%) were prospectively followed until a corrected age of 24 months. At birth, infants with lowest-quartile TSH had higher NDI risks (odds ratio [OR] 2.3; 95% confidence interval [CI], 1.3–4.1, *P* = 0.004) compared to infants with interquartile-range TSH. Conversely, by TEA/discharge, infants with highest-quartile TSH had increased NDI (OR 1.9; 95% CI, 1.0–3.4, *P* = 0.03). By paired TSH categories, infants persistently in the lowest TSH quartile (48%; aOR 4.4; 95% CI, 1.4–14.5, *P* = 0.01) and those with a shift from interquartile range to the highest quartile (32%; aOR 2.7; 95% CI, 1.0–7.4, *P* = 0.046) had increased NDI risks compared with the reference with consistent interquartile-range TSH.

**Conclusion:**

Extremely preterm infants persistently in the lowest-quartile TSH level at birth and at discharge had the highest NDI risk. TSH quartile levels measured during NBS may serve as a population surrogate biomarker for assessing NDI risks in infants born extremely preterm.

## INTRODUCTION

Despite the improvement in survival rate, infants born extremely preterm continue to experience a high proportion of neurodevelopmental impairment (NDI) at follow-up, including cerebral palsy, hearing/visual impairment, and delayed motor and cognitive development, which could greatly impact children’s long-term quality of life.^1^^,^^2^ Sophisticated biomarkers, such as advanced magnetic neuroimaging or continuous electroencephalogram monitoring, have been applied for outcome prediction in preterm infants.^3^^–^^6^ However, these tools are available only in research settings, and a population-level biomarker for NDI risks in extremely preterm infants is still lacking.

Newborn screening (NBS) programs have been implemented at population level to facilitate early diagnosis of inherited metabolic disorders including congenital hypothyroidism, for which the primary thyroid-stimulating hormone (TSH) screening strategy has been widely adopted.^7^ Congenital hypothyroidism, a disorder of thyroid hormone deficiency due to incomplete thyroid development or inadequate thyroid hormone production, is regarded as the leading cause of preventable intellectual disability in children.^7^ In hypothyroid status, the pituitary gland releases more TSH as regulated by thyrotropin-releasing hormone from the hypothalamus, to stimulate thyroxine secretion by the thyroid gland.^7^ Taking advantage of the feedback loop of hypothalamus-pituitary-thyroid axis, a cut-off value defining elevated TSH has been applied in NBS worldwide, to identify neonates at risk of congenital hypothyroidism early for prompt thyroxine supplementation in confirmed cases, subsequently preventing sequelae of intellectual disability.^7^^,^^8^

In preterm infants, while most studies focus on high TSH levels suggesting hypothyroid status, the significance of low TSH levels remains unclear. In general, low TSH levels may suggest hyperthyroidism, but hyperthyroidism in neonates is rare and mostly associated with maternal thyroid disorders that can be differentiated from the history.^9^ The hypothalamus-pituitary-thyroid axis is immature in extremely preterm infants, leading to a more intricate interpretation for TSH levels from NBS.^7^ Our previous study demonstrated that extremely preterm infants who had lowest-quartile TSH levels by the first NBS conducted at 24–96 hours after birth were associated with lung and brain complications in the neonatal intensive care units (NICUs),^10^ suggesting low TSH levels as a potential marker for brain dysfunction. However, the significance of low TSH levels for neurodevelopmental outcomes at long-term follow-up remains unclear. A clinical trial study of daily iodine supplementation in very preterm infants <31 weeks of gestation who had blood sampling on postnatal days 7, 14, and 28 and postmenstrual age 34 weeks showed that infants consistently in the top decile TSH levels had decreased cognitive and motor scores assessed using the Bayley Scales of Infant and Toddler Development (BSID), Third Edition (BSID-III) at age 2 years compared with those not in the top decile.^11^ In contrast, a single center study from Wisconsin enrolling very preterm infants <32 weeks of gestation did not identify an association between TSH percentiles by the NBS obtained at postmenstrual age 37 weeks and BSID-III neurodevelopmental outcome scores at corrected age 18–22 months.^12^ Therefore, whether TSH can be a useful biomarker for NDI risks in infants born extremely preterm remains unclear.

In Taiwan, NBS for preterm infants includes the first screening sampled within the first few postnatal days and a second screening performed at term-equivalent age (TEA) or upon discharge. The NBS program in Taiwan uses primary-TSH-with-backup-thyroxine method, so the database contains only TSH levels. Thyroid function tests, including thyroxine levels, are examined in detail at referral hospitals for those with high TSH assessed during NBS. Using an extremely preterm population cohort enrolling infants born <29 weeks of gestation from a geographically defined area in southern Taiwan, the study hypothesized that the quartile levels of TSH measured during NBS at birth and at TEA/discharge are associated with different risks of NDI outcomes at follow-up in extremely preterm infants.

## METHODS

### Participants

Infants born <29 weeks of gestation in 2008–2020 and admitted to all the NICUs in Tainan City were enrolled. The city, with a population of nearly 2 million people in southern Taiwan, is served by three regional hospitals, one tertiary center, and one academic tertiary center of the university hospital for the intensive care of preterm neonates. Infants whose mothers had thyroid disorders, such as hyperthyroidism or hypothyroidism, and infants who required thyroxine supplementation during hospitalization were excluded.

In addition, the TSH levels measured during NBS of all term neonates born at gestational age ≥37 weeks in the city in one randomly-selected month during the study period were obtained for comparison.

The study was approved by the review board of National Cheng Kung University Hospital (ER-98-135). Parents signed the consents for data collection when infants were hospitalized in the NICUs and at neurodevelopmental follow-up visits.

### TSH measurement by NBS program for infants born preterm

The national NBS program for congenital hypothyroidism in Taiwan utilizes the primary-TSH-with-backup-thyroxine method. Since 2008, in addition to the screening performed in the early postnatal days, preterm infants have received additional TSH screening at TEA/discharge. Therefore, preterm infants included in this study underwent TSH screening tests at two time points: at birth (24–96 hours of age) and at TEA/discharge (postmenstrual age 35–44 weeks).

Whole-blood TSH levels in the dried blood spot specimens were measured using a time-resolved fluoroimmunoassay (AutoDELFIA kit of neonatal human TSH, Perkin Elmer, Turku, Finland) at the NBS Center of the Chinese Foundation of Health, a semi-government non-profit organization for NBS program in Taiwan. Blood TSH levels ≥40 µU/mL were considered positive for congenital hypothyroidism. Infants with TSH levels ≥10 µU/mL but <40 µU/mL were classified as suspected congenital hypothyroidism necessitating re-examinations. Blood TSH levels <10 µU/mL were considered normal. Infants whose TSH levels ≥10 µU/mL were referred to pediatric endocrinologists for further evaluations.^7^^,^^13^ In Taiwan, the same cutoff value applies to infants born at term and preterm, and for early neonatal and TEA/discharge screenings.

### Neonatal morbidities in NICUs

Demographic and perinatal data, including the use of dopamine due to its potential interaction with TSH secretion,^14^ were recorded. Neonatal morbidities were reviewed, including severe brain injury (grade III intraventricular hemorrhage with ventriculomegaly and/or intraparenchymal hemorrhage, and cystic periventricular leukomalacia), necrotizing enterocolitis (NEC) stage ≥2a,^15^ hemodynamically-significant patent ductus arteriosus (hs-PDA) requiring surgical interventions, bronchopulmonary dysplasia (BPD; defined as oxygen treatment >21% for at least 28 days followed by an assessment at 36 weeks of postmenstrual age or discharge),^16^ and severe retinopathy of prematurity (ROP; defined as stage ≥2 plus disease or requiring treatment in either eye).^17^

### Neurodevelopmental outcomes

Neurodevelopmental outcomes were evaluated at a corrected age of 24 months by a team of pediatric neurologists and child psychologists at the university hospital, who were blinded to the TSH data when performing assessments. Infants born before 2011 were assessed using the BSID, Second Edition (BSID-II), and those born after 2011 were evaluated using BSID-III. Cerebral palsy was defined as abnormal muscle tonicity and gross motor function limitations with a gross motor function classification system score ≥2. Hearing impairment referred to permanent hearing loss that affected the ability to understand or communicate, or with a threshold higher than 55 dB in the better ear. Visual impairment represented bilateral blindness. Cognitive delay was defined as a mental developmental index <70 using BSID-II or a cognitive composite score <85 using BSID-III, and motor delay was defined as a psychomotor developmental index <70 using BSID-II or a motor composite score <85 using BSID-III.^18^ NDI encompassed any of the following deficits: cerebral palsy, hearing/visual impairment, or delayed cognitive or motor development.^19^^,^^20^

### Statistical analyses

Density plots were used to present TSH distribution levels. The Wilcoxon signed-rank test was employed to compare the paired TSH data collected at birth and at TEA/discharge, and the Mann-Whitney test was used to compare the TSH data of preterm infants at TEA/discharge with those of term neonates. Clinical characteristics of infants with and without neurodevelopmental follow-up were compared using Fisher’s exact test for categorical variables and the Mann-Whitney test for continuous variables. TSH levels obtained through NBS at birth and at TEA/discharge were respectively divided into three groups based on quartiles: the lowest quartile, the interquartile range, and the highest quartile, according to previous literatures of thyroid function tests on neuropsychiatric outcomes.^21^ The associations of NDI with different TSH quartile groups during screening at birth, TEA/discharge screening, and paired TSH quartile patterns were first examined using univariable logistic regression models, followed by multivariable logistic regression model adjusted for clinical factors that might associate with NDI, including gestational age, sex, maternal educational status, use of dopamine, total counts of neonatal morbidities, and the year of birth. For the quartile groups categorized by paired TSH data that showed increased odds for NDI, univariable logistic regression models were used to examine their associations with individual neonatal morbidity. The significance level was set at *P* < 0.05 for 2-sided hypothesis tests. The statistical analyses were performed using GraphPad Prism 9 (GraphPad Software, Inc, San Diego, CA, USA) and R version 4.0.2 (R Foundation for Statistical Computing, Vienna, Austria).

## RESULTS

### Enrollment of participants

**Figure 1.**
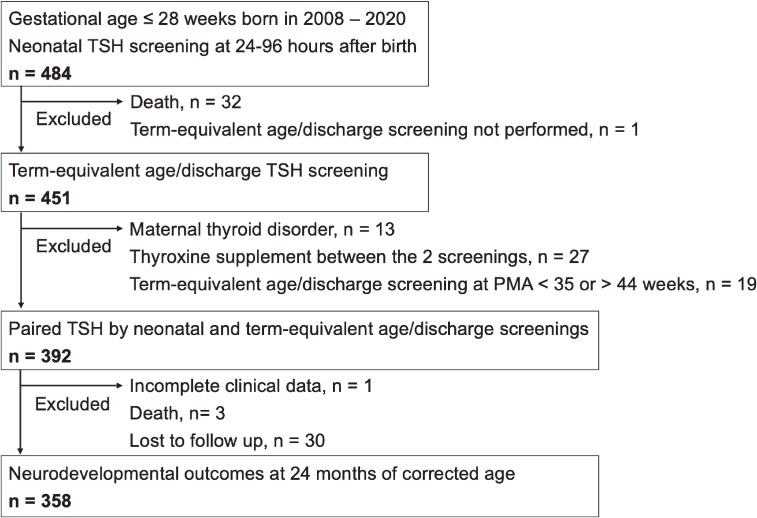
Flowchart of case enrollment.

A total of 484 infants with extremely preterm birth underwent NBS for TSH at 24–96 hours of age. After excluding 32 infants who died during hospitalization and 1 infant whose TEA/discharge screening was not conducted, 451 infants had TSH screening at TEA/discharge. Infants excluded from analysis encompassed 13 infants whose mothers had a thyroid disorder, 27 infants who received thyroxine supplementation between the first and second TSH screening, and 19 infants whose TEA/discharge screening tests were performed earlier than 35 or later than 44 weeks of postmenstrual age. Among the remaining 392 infants, three patients died after discharge, one patient did not have complete clinical information for analysis, and 30 patients were lost to follow-up (Figure 1).

### Distribution of TSH data in preterm and term infants

**Figure 2.**
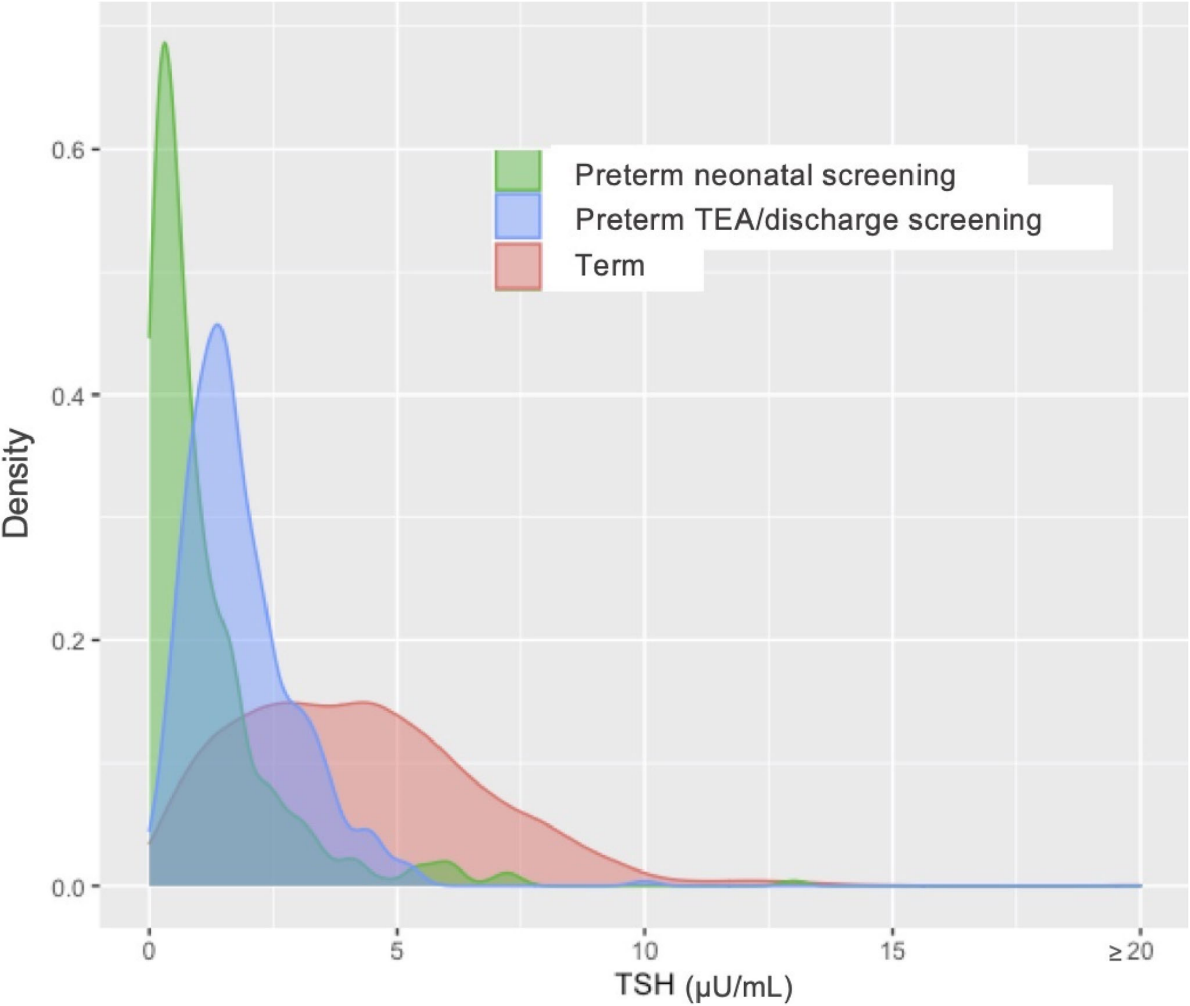
Density plots of TSH levels by NBS in infants born extremely preterm and at term. Preterm infants: early neonatal screening at 24–96 hours after birth (green) and term-equivalent age (TEA)/discharge screening at postmenstrual age 35–44 weeks (blue). Term neonates: newborn screening at 48–72 hours of age (red).

The density plots display the TSH levels obtained through NBS at birth and at TEA/discharge from the 392 infants born preterm, in comparison to the NBS data from 1,788 neonates born at term (Figure 2). In preterm infants, the TSH levels at TEA/discharge (1^st^ quartile: 1.1 µU/mL, median: 1.6 µU/mL, and 3^rd^ quartile: 2.4 µU/mL) were higher than the levels obtained at birth (1^st^ quartile: 0.3 µU/mL, median: 0.6 µU/mL, 3^rd^ quartile: 1.5 µU/mL; *P* < 0.001). Even when preterm infants reached TEA, their TSH levels remained significantly lower than the levels of term neonates (1^st^ quartile: 2.3 µU/mL, median: 4.0 µU/mL, and 3^rd^ quartile: 5.7 µU/mL; *P* < 0.001).

### Clinical characteristics and neurodevelopmental outcomes

A total of 358 (91%) infants who had neurodevelopmental outcomes were included in the analysis. The 30 infants lost to follow-up had larger birth weights and lower rates of multiple gestation or receiving prenatal steroids compared to the infants who underwent follow-up assessments (Table 1). The 358 infants were born at a median gestation of 27 weeks, with a median birth weight of 956 grams, and were composed of 203 (57%) male and 155 (43%) female infants. Among them, 66 (18%) infants were diagnosed with severe brain injury (Table 2). NDI was observed in 82 (23%) infants, including 20 (6%) of cerebral palsy, 8 (2%) of hearing impairment, 1 (0.3%) of visual impairment, 57 (16%) of cognitive delay, and 55 (15%) of motor delay. After the NBS at TEA/discharge, 3 (0.8%) infants received thyroxine supplementation.

**Table 1.** Differences in demographic and perinatal data between infants with follow-up assessment and those lost to follow-up

	Follow-up *N* = 358	Lost to follow-up *N* = 30	*P*-value
Gestational age, weeks, median (IQR)	27 (2)	27 (2)	0.4
Birth weight, grams, median (IQR)	956 (330)	1,024 (221)	0.02
Male, *N* (%)	203 (57)	19 (63)	0.6
Maternal education below college level, *N* (%)	188 (53)	18 (60)	0.5
Small for gestational age, *N* (%)	20 (6)	1 (3)	0.7
Multiple gestation, *N* (%)	85 (24)	2 (7)	0.04
Preeclampsia, *N* (%)	62 (17)	3 (10)	0.4
Chorioamnionitis, *N* (%)	52 (15)	5 (17)	0.8
Prenatal steroids, *N* (%)	313 (87)	20 (67)	0.005
5-minute Apgar score, median (IQR)	8 (1)	8 (1)	0.2
Dopamine usage during hospitalization, *N* (%)	192 (54)	16 (53)	1.0

IQR, interquartile range.

**Table 2.** Neonatal morbidities and neurodevelopmental outcomes of 358 extremely preterm infants who received neurodevelopmental assessments at follow-up

Neonatal morbidities	*N* (%)
Severe brain injury	66 (18)
Necrotizing enterocolitis stage ≥IIa	34 (9)
Hemodynamically-significant patent ductus arteriosus requiring surgical intervention	57 (16)
Bronchopulmonary dysplasia	154 (43)
Severe retinopathy of prematurity	59 (16)

Neurodevelopmental outcomes	*N* (%)

Cerebral palsy with GMFCS score ≥2	20 (6)
Hearing/Visual impairment	8/1 (2/0.3)
Cognitive delay	57 (16)
Motor delay	55 (15)
Neurodevelopmental impairment^a^	82 (23)

GMFCS, gross motor function classification system.

^a^Neurodevelopmental impairment included any of the following deficits: cerebral palsy, hearing/visual impairment, or delayed cognitive or motor development.

### NDI risks by TSH quartiles of NBS at birth and at TEA/discharge

The TSH data obtained through NBS at birth and at TEA/discharge periods were categorized into the lowest quartile, the interquartile range, and the highest quartile. Using the group with TSH in the interquartile range as reference, we examined the NDI risks of infants in the lowest and highest quartiles of TSH. Based on NBS at birth, infants with TSH in the lowest quartile had higher odds of NDI compared with the reference group (37% vs 20%; odds ratio [OR] 2.3; 95% confidence interval [CI], 1.3–4.1, *P* = 0.004) (Table 3). Conversely, based on NBS at TEA/discharge, infants with TSH in the highest quartile had higher odds of NDI compared with reference (30% vs 19%; OR 1.9; 95% CI, 1.0–3.4, *P* = 0.03).

**Table 3.** Neurodevelopmental impairment rates by TSH quartiles of newborn screening at birth and at term-equivalent age/discharge

TSH quartiles	Whole-blood TSH ranges, µU/mL	Infants, *N*	Neurodevelopmental impairment, *N* (%)	Odds ratio (95% CI)	*P*-value
**At-birth screening**					
Lowest quartile	TSH <0.3	84	31 (37)	2.3 (1.3–4.1)	0.004
Interquartile	0.3≤ TSH <1.5	184	37 (20)	Reference	—
Highest quartile	TSH ≥1.5	90	14 (16)	0.7 (0.4–1.4)	0.4
**Term-equivalent age/discharge screening**					
Lowest quartile	TSH <1.1	88	21 (24)	1.4 (0.7–2.5)	0.3
Interquartile	1.1≤ TSH <2.4	177	33 (19)	Reference	—
Highest quartile	TSH ≥2.4	93	28 (30)	1.9 (1.0–3.4)	0.03

CI, confidence interval; TSH, thyroid-stimulating hormone.

### NDI risks by patterns of paired TSH quartiles

**Figure 3.**
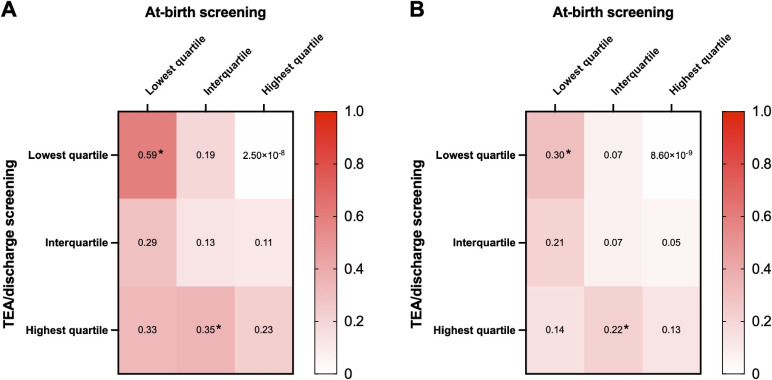
Heatmap of predicted probabilities of NDI by paired TSH quartile groups and sex. Median predicted probabilities of NDI by TSH quartile groups were adjusted for gestational age, sex, use of dopamine, maternal educational status, total counts of neonatal morbidities, and the year of birth. Male infants **(A)** had higher predicted probabilities of NDI than female infants **(B)**. No infant with TSH transiting from the highest quartile at birth to the lowest quartile at TEA/discharge had NDI. ^*^Statistically significant compared with the reference group with consistent interquartile-range TSH levels.

We then examined the NDI risks based on the patterns of paired TSH quartiles at the two time points. Using infants who had interquartile-range TSH during both NBS at birth and at TEA/discharge as the reference, we found that infants with TSH in the lowest quartile at birth followed by any quartile at TEA/discharge were more susceptible to NDI (lowest to lowest quartile: 48%; OR 5.2; 95% CI, 1.9–14.6, *P* = 0.001; lowest quartile to interquartile range: 32%; OR 2.7; 95% CI, 1.1–6.4, *P* = 0.03; lowest to highest quartile: 35%; OR 3.1; 95% CI, 0.9–9.8, *P* = 0.05) (Table 4). Infants who had TSH in the interquartile range during NBS at birth but increased to the highest quartile at TEA/discharge also had higher odds of NDI (32%; OR 2.7; 95% CI, 1.1–6.4, *P* = 0.03). After adjusting for gestational age, dopamine usage, sex, maternal education below college level, total counts of neonatal morbidities, and the year of birth, infants whose TSH persistently in the lowest quartile (adjusted OR 4.4; 95% CI, 1.4–14.5, *P* = 0.01) and those with a shift from interquartile range to the highest quartile (adjusted OR 2.7; 95% CI, 1.0–7.4, *P* = 0.046) continued to have increased odds of NDI. In addition, male infants (adjusted OR 2.0; 95% CI, 1.1–3.9, *P* = 0.03), higher counts of neonatal morbidities (adjusted OR 1.9; 95% CI, 1.4–2.6, *P* < 0.001), and the year of birth (less NDI in infants born in 2009, 2012, and 2019; reference: 2008) were also associated with increased NDI. The predicted probabilities for developing NDI by the multivariable regression model are presented in Figure 3.

**Table 4.** Risks for neurodevelopmental impairment by the paired quartile patterns of TSH

Paired TSH quartile patterns	Infants, *N*	Neurodevelopmental impairment, *N* (%)	Odds ratio (95% CI)	*P*-value	Adjusted odds ratio^a^ (95% CI)	*P*-value
**At-birth screening: lowest quartile**						
***TEA/discharge screening***						
Lowest quartile	23	11 (48)	5.2 (1.9–14.6)	0.001	4.4 (1.4–14.5)	0.01
Interquartile	44	14 (32)	2.7 (1.1–6.4)	0.03	1.7 (0.6–4.8)	0.3
Highest quartile	17	6 (35)	3.1 (0.9–9.8)	0.05	2.0 (0.5–7.5)	0.3
**At-birth screening: interquartile**						
***TEA/discharge screening***						
Lowest quartile	53	10 (19)	1.3 (0.5–3.3)	0.5	1.4 (0.5–3.8)	0.5
Interquartile	87	13 (15)	Reference	—	Reference	—
Highest quartile	44	14 (32)	2.7 (1.1–6.4)	0.03	2.7 (1.0–7.4)	0.046
**At-birth screening: highest quartile**						
***TEA/discharge screening***						
Lowest quartile	12	0 (0)	NA^b^	—	NA^b^	—
Interquartile	46	6 (13)	0.9 (0.3–2.3)	0.8	0.7 (0.2–2.4)	0.6
Highest quartile	32	8 (25)	1.9 (0.7–5.1)	0.2	1.5 (0.5–4.7)	0.5

CI, confidence interval; NA, not available; TEA, term-equivalent age; TSH, thyroid-stimulating hormone.

^a^Adjusted for gestational age, sex, use of dopamine, maternal education below college level, total counts of neonatal morbidities, and the year of birth.

^b^No infant had neurodevelopmental impairment in this group.

### TSH quartile patterns and neonatal morbidities

**Figure 4.**
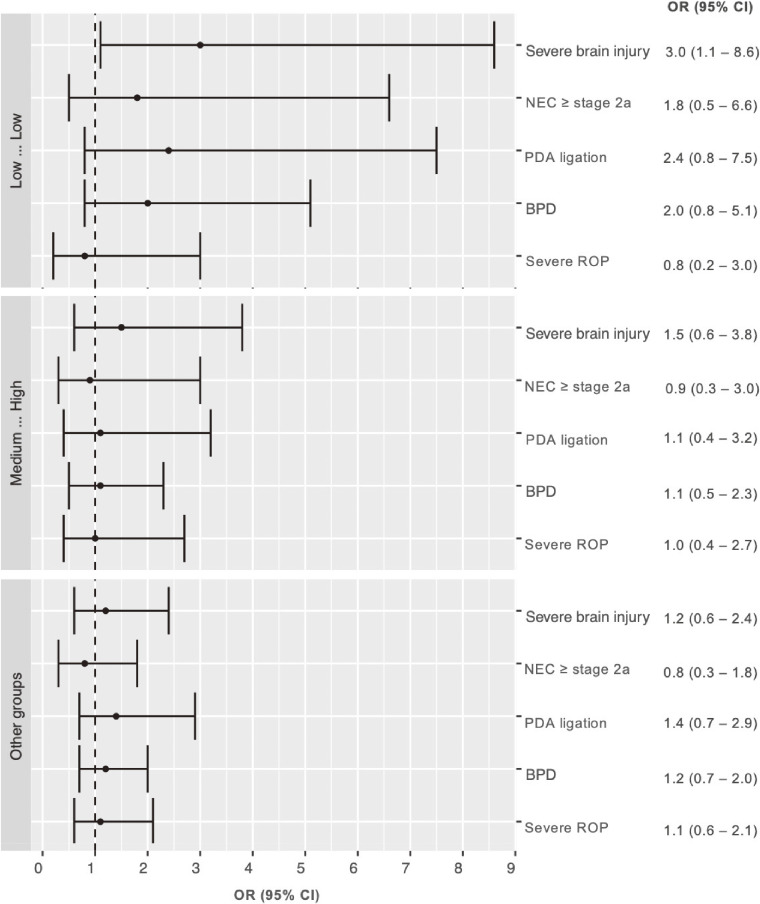
Neonatal morbidities and paired TSH quartile patterns. Reference: TSH in the interquartile range by both NBS at birth and at TEA/discharge. Low – Low: TSH persistently in the lowest quartile. Medium – High: TSH shifting from the interquartile range to the highest quartile. Other groups: the remaining 6 TSH patterns.

We further analyzed whether the paired TSH quartile patterns with increased NDI risks were correlated with any specific neonatal morbidities. Compared with the reference group, infants with TSH persistently in the lowest quartile were associated with severe brain injury, whereas infants whose TSH increased from interquartile range to the highest quartile and other groups did not show an association with any specific neonatal morbidity (Figure 4). When adjusted for severe brain injury, NDI remained significantly increased in infants whose TSH persistently stayed in the lowest quartile (aOR 4.1; 95% CI, 1.3–13.3, *P* = 0.02).

## DISCUSSION

### Main findings

In the population cohort of infants born extremely preterm, the risk of NDI at 24 months of corrected age was associated with the changing patterns of TSH quartiles assessed during NBS from birth to TEA/discharge. The cohort used TSH data obtained through routine NBS program as a population-level marker for NDI. Infants enrolled in the study were followed up prospectively, with a follow-up rate of more than 90% at 24 months of corrected age. Our findings indicate that, compared to infants with consistent interquartile-range TSH, infants with TSH persistently in the lowest quartile had a 4-fold risk, and those whose TSH elevated from interquartile range to the highest quartile had a nearly 3-fold risk of NDI. These results suggest that TSH obtained through routine NBS is useful to stratify NDI risks, allowing for early identification and potential early intervention in high-risk infants.

### NDI markers in preterm infants

NDI is one of the most concerned outcomes in extremely preterm infants.^22^ Predicting NDI risks at discharge may lead to earlier initiation of interventions, which can be more effective in alleviating impairments compared to initiating interventions at school age.^23^ Several models have been proposed to predict NDI in clinical settings for preterm populations, mostly relying on a combination of physiological profiles at admission and neonatal morbidities before discharge.^22^ Neuroimaging and circulating neural biomarkers have further advanced the prediction of NDI at research level.^24^^–^^27^ As biomarkers used at population level to forecast NDI in extremely preterm infants are rarely reported, we took advantage of the NBS database and proposed TSH levels as a potential early marker for NDI.

### NBS for mortality and morbidities at acute stage in NICUs

NBS, initially used to identify neonates at risk of inherited metabolic disorders, has started to contribute as metabolomic profiles in evaluating mortality and major morbidities associated with preterm birth in NICUs,^10^^,^^28^^,^^29^ but it’s association to neurodevelopmental outcomes at follow-up stage has not been established before.^12^ Our study extended the application of NBS beyond the acute stage in NICUs and into follow-up stage at 24 months of corrected age, validating the value of NBS in forecasting neurodevelopmental outcomes in the extremely preterm population.

### Vulnerability of the immature hypothalamus-pituitary-thyroid axis in preterm infants

Extremely preterm infants are born during the critical window when TSH secretion is not fully coordinated by upstream or downstream regulatory/feedback hormones.^30^ Our results showed that, even when these extremely preterm infants reached TEA, the density plot of TSH distribution at TEA/discharge still differed significantly from the density plot of term babies, suggesting that the extrauterine maturation process experienced by extremely preterm infants is not equal to the intrauterine maturation process in full-term pregnancy.

Critical illnesses and neonatal morbidities may further complicate the maturation process of the axis.^31^ We found that infants with TSH persistently in the lowest quartile, in whom nearly half of them had NDI at a corrected age of 24 months, were associated with severe brain injuries. The mechanism underling neuroendocrine dysfunction during or after severe brain injuries remains unknown, but may involve neuroinflammation, vasospasm and hypoperfusion, and increased intracranial pressure that affects the hypothalamohypophysial portal blood supply.^32^^–^^35^ However, even after adjusting for severe brain injury, we still observed a 4-fold risk of NDI, suggesting that the association between persistently low TSH levels and NDI may not be fully mediated through the presence of severe brain injury.

### Low TSH and brain function alteration

Hypothalamus-pituitary-thyroid axis plays an important role in the brain function of children and adults.^21^^,^^36^^–^^39^ For example, in a study enrolling nearly 30,000 adult subjects, low TSH levels were correlated to clinically relevant depression irrespective of subclinical or overt hyperthyroidism/hypothyroidism, suggesting low TSH levels as a marker for altered mental function independent of thyroxine levels.^38^ According to a population cohort in California enrolling mostly term babies, infants with a pattern of lower neonatal TSH levels were at higher risk for developmental delay.^21^ In addition, autistic children with delayed social-emotional development were shown to exhibit decreased TSH secretion upon thyrotropin-releasing hormone stimulation compared with controls.^39^ Therefore, our findings of increased NDI risks in extremely preterm infants with persistent low TSH levels may illustrate a blunted response of pituitary gland to the upstream hypothalamic control in the situation of deviated neurodevelopment.^39^

### Increasing TSH and NDI risks

Previous studies have focused on the relationship between mildly elevated TSH levels suggesting subclinical hypothyroidism and neurodevelopmental outcomes in preterm infants. An iodine supplementation trial examining serial TSH levels collected on postnatal days 7, 14, and 28 and at postmenstrual age 34 weeks during hospitalization showed that infants with persistently high TSH levels in the top decile had inferior scores in cognition and motor function.^11^ However, the design of a clinical therapeutic trial and differences in laboratory techniques for TSH measurements across different hospitals may complicate the generalization of their findings. Interpreting NBS TSH data for outcome correlation could be more accessible, as it covers all preterm graduates from NICUs since NBS programs conduct nationwide screening using uniform techniques with limited trials of sampling.

Our study excluded infants with thyroid dysfunction necessitating thyroxine supplementation, and we still noticed that infants with an increasing pattern of TSH from interquartile range to the highest quartile had a higher risk for NDI, without an association to any specific neonatal morbidity. Previous studies have found that increased TSH levels but without thyroxine deficiency are more common in preterm and low-birth-weight infants.^40^^,^^41^ This phenomenon might be related to fetal growth restriction that changes the amount and isoform of hormone receptors in the immature brain and alters the function of the hypothalamus-pituitary-thyroid axis.^40^ The clinical significance of this rising TSH pattern remains unclear,^42^ but an association with neurodevelopment was suggested by our findings. Further investigations are required to understand the mechanism linking isolated increased TSH concentration and neurodevelopmental outcomes in infants born extremely preterm.

### Limitations

Limitations of this study include the lack of thyroxine data for infants with normal-range TSH levels according to the primary-TSH-with-backup-thyroxine NBS strategy. Therefore, we cannot further explore the role of thyroxine deficiency or excessiveness in NDI among infants with normal-range TSH at NBS.^43^ Additionally, the study included a relatively small number of cases despite being derived from a population study, which was limited by the low rate of extremely preterm birth affecting 2–5 per 1,000 pregnancies.^44^ The low incidence of extremely preterm birth restricted this study from examining TSH levels in more details. Therefore, the generalizability of our findings may be limited because a proper cutoff value to define low or high TSH is not available. Therefore, recruiting a larger number of infants born extremely preterm with neurodevelopmental follow-up data is necessary to validate the association between the changing patterns of TSH levels from early postnatal period to TEA and NDI risks.

In conclusion, extremely preterm infants persistently in the lowest-quartile TSH level at birth and at discharge had the highest risk of NDI at follow-up. The TSH quartile levels determined through NBS may serve as a population surrogate biomarker for assessing the risk of NDI in infants born extremely preterm, aiding neurodevelopmental monitoring in targeted subgroups of infants for potential early intervention.
